# Circulating histones contribute to monocyte and MDW alterations as common mediators in classical and COVID-19 sepsis

**DOI:** 10.1186/s13054-022-04138-2

**Published:** 2022-08-30

**Authors:** Daniela Ligi, Bruna Lo Sasso, Rosaria Vincenza Giglio, Rosanna Maniscalco, Chiara DellaFranca, Luisa Agnello, Marcello Ciaccio, Ferdinando Mannello

**Affiliations:** 1 Unit of Clinical Biochemistry, Section of Biochemistry and Biotechnology, Department of Biomolecular Sciences -DISB, University of Urbino Carlo Bo, Via A. Saffi, 2, 61029 Urbino, Italy; 2 Institute of Clinical Biochemistry, Clinical Molecular Medicine and Clinical Laboratory Medicine, Department of Biomedicine, Neurosciences and Advanced Diagnostics, BiND, University of Palermo, Via del Vespro, 90127 Palermo, Italy; 3 Department and U.O.C. Laboratory Medicine, University Hospital “Paolo Giaccone” of Palermo, Palermo, Italy

**Keywords:** Histones, COVID-19, Sepsis, Critical care, Monocyte distribution width, Monocyte, Biomarkers

## Abstract

**Objective:**

Histone proteins are physiologically involved in DNA packaging and gene regulation but are extracellularly released by neutrophil/monocyte extracellular traps and mediate thrombo-inflammatory pathways, associated to the severity of many human pathologies, including bacterial/fungal sepsis and COVID-19. Prominent and promising laboratory features in classic and viral sepsis emphasize monocyte distribution width (MDW), due to its ability to distinguish and stratify patients at higher risk of critical conditions or death. No data are available on the roles of histones as MDW modifiers.

**Design:**

Comparison of MDW index was undertaken by routine hematology analyzer on whole blood samples from patients with COVID-19 and Sepsis. The impact of histones on the MDW characteristics was assessed by the in vitro time-dependent treatment of healthy control whole blood with histones and histones plus lipopolysaccharide to simulate viral and classical sepsis, respectively.

**Measurements and main results:**

We demonstrated the breadth of early, persistent, and significant increase of MDW index in whole blood from healthy subject treated in vitro with histones, highlighting changes similar to those found in vivo in classic and viral sepsis patients. These findings are mechanistically associated with the histone-induced modifications of cell volume, cytoplasmic granularity and vacuolization, and nuclear structure alterations of the circulating monocyte population.

**Conclusions:**

Histones may contribute to the pronounced and persistent monocyte alterations observed in both acute classical and viral sepsis. Assessment of the biological impact of circulating histone released during COVID-19 and sepsis on these blood cells should be considered as key factor modulating both thrombosis and inflammatory processes, as well as the importance of neutralization of their cytotoxic and procoagulant activities by several commercially available drugs (e.g., heparins and heparinoids).

**Supplementary Information:**

The online version contains supplementary material available at 10.1186/s13054-022-04138-2.

## Introduction

Histones are key components for chromatin physiological functions but extracellularly mobilized during pathological processes [1]. Acting as endogenous damage-associated molecular pattern molecules, histones mediate both inflammatory pathways and coagulative cascade linked to the severity of several pathologies, including Sepsis and COVID-19 [2–6]. In fact, histones interact with blood cells (e.g., monocytes and platelets) promoting cytotoxicity, inducing phosphatidylserine exposure, modulating Toll-like receptors, releasing pro-inflammatory cytokine/chemokines and activating the coagulative cascade [1].

Laboratory findings in COVID-19 diagnosis and prognosis [7, 8] highlighted that leukocyte extracellular traps (including histones, extracellular DNA, oxidant and proteolytic enzymes) emerged as diagnostic/prognostic markers in COVID-19 [3, 9], actively participating in both cytokine storm and coagulation dysfunctions [1]. Recently, circulating histones were emphasized as predictive biomarkers [1] in patients with severe COVID-19 “viral Sepsis” [10], acting as sub-lethal signaling molecules and inducing cytokine storm [9].

Among common laboratory biomarkers shared by SARS-CoV-2 and Sepsis infections [7, 11], the modification of the hematological parameter Monocyte Distribution Width (MDW) predicts both multiorgan failure and increased mortality rate in Sepsis conditions [11]. MDW index (FDA-approved, EC-marked early Sepsis indicator of monocyte heterogeneity upon massive inflammatory activation [11]) is further recognized as diagnostic/prognostic marker for COVID-19 severity and clinical outcomes, as a kind of novel viral Sepsis biomarker [12–14].

Researches had linked MDW index to both COVID-19 and Sepsis [11], as well as studies have associated Sepsis and COVID-19 to histone levels [6, 15]; curiously, no data are currently available on histones as MDW modifiers. These bases raised the possibility that histones may contribute to the activation and morphological dysregulation of monocytes in both COVID-19 and Sepsis infections [4, 16]. With our whole blood in vitro model, we investigated the ability of histones to modify monocyte morphology and MDW index. We further compared these in vitro modifications to those measured in COVID-19 and Sepsis patients.

## Materials and methods

Healthy subjects were voluntarily recruited among staff at the Dept BiND of University of Palermo and Dept DISB of University of Urbino. MDW values and clinical data for both COVID-19 (*n* = 7, age range 52–85 years) and Sepsis (*n* = 8, age range 47–81 years) patients were extracted by data archives of the University Hospital of Palermo.

The cut-offs reported for both MDW index and histone values are in agreement with literature references (healthy control subjects [5, 6, 11, 12, 15]; COVID-19 patients [12, 14, 15]; and Sepsis patients [5, 6, 11]).

Our non-interventional in vitro study was in accordance to the Declaration of Helsinki principles, peripheral venous whole EDTA blood samples were collected from healthy volunteers (*n* = 6, mean age 48.5 ± 15 years, range 31–63 years). Routine complete blood cell counts were performed on *UniCell DxH900 Hematology Analyzer* (Beckman Coulter). Automated slide preparation (*unit SP-100, DI-60 system workflow*, Sysmex) was used to obtain May-Grunwald-Giemsa-stained blood smears.

### Statistical analyses

All statistical tests were performed using GraphPad Prism 9.0. Values are expressed as mean ± standard error (SEM) and *p* values < 0.05 were considered significant. Unless otherwise specified, significant differences between groups were determined using one-way ANOVA followed by post-hoc test (i.e., Tukey’s multiple comparison test). Regression analyses were performed through simple linear regression.

## Results

**Fig. 1:**
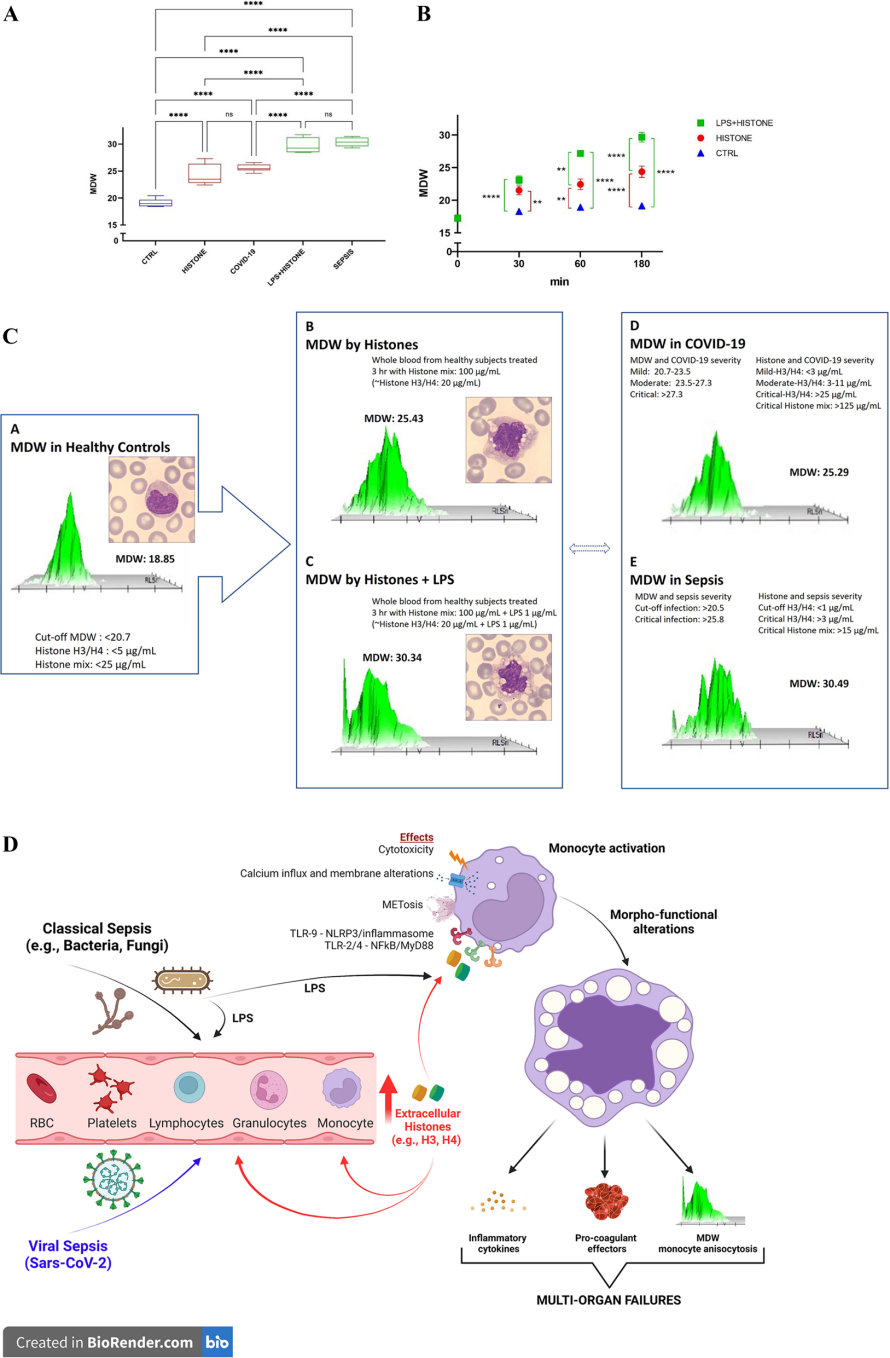
MDW index modifications in whole EDTA blood samples collected from healthy subjects treated in vitro with 100 µg/mL of histone mixture and 100 µg/mL of histone mixture + 1 µg/mL of LPS, compared to COVID-19 and Sepsis profiles, and mechanistic network of histone actions in sepsis. Sepsis patients was categorized according to Sepsis-2/3 diagnostic criteria; *n* = 8, mean age 63 ± 13.2 years; median SOFA score of 3, range 2–7; no patient needed for mechanical ventilation or continuous renal replacement therapy. COVID-19 patients had mild/moderate SARS-CoV-2 infection; *n* = 7, mean age 68 ± 14.4 years; no patient needed for mechanical ventilation. Aliquots of 1 mL of whole blood from each volunteer were exposed to a mixture of commercially available histones (100 µg/mL) (Histone from calf thymus, Sigma), in absence or presence of 1 µg/mL of lipopolysaccharide (LPS) (from Escherichia coli O127:B8 strain, Sigma). The samples, maintained at RT, were analyzed for MDW at 30, 60 and 180 min after careful inversion avoiding sedimentation of blood cells, and processed within 4 h of collection. MDW and routine complete blood cell counts were performed on *UniCell DxH900 Hematology Analyzer* (Beckman Coulter). The choice of whole blood treatment with 100 µg/mL of a mixture of commercially available human histones is in agreement with the literature evidence suggesting that the concentration of 20 µg/mL of circulating histone H3 was detected in patients with critical COVID-19 [15] and that the same deleterious effects of histone H3 is obtained with five-fold higher concentrations of mixture of histones [3]. The MDW values, scatter plots and blood smears are representative of at least triplicate analyses. Values are plotted as mean ± SEM (**very significant = *p: *0.001–0.01; ****extremely significant = *p* < 0.0001)*.* **A** MDW modifications after histones and LPS + histone treatments for 3 h in control subjects compared with classical and viral Sepsis. **B** Time-dependent increases of MDW values (linear regressions: control subjects, *Y* = 0.01029*x* + 17.45* r*^2^ = 0.4065; histone 100 µg/mL, *Y* = 0.03751*x* + 18.06 *r*^2^ = 0.6995; 100 µg/mL of histone mixture + 1 µg/mL of LPS, *Y* = 0.06951*x* + 17.85* r*^2^ = 0.9317). **C** Representative modifications of MDW, blood smears and scatter plots in both classical and viral Sepsis, compared to histone and histone + LPS whole blood treatments. **D** Schematic representation of a possible predictive/mechanistic network of how circulating histones commonly mediate monocyte alterations in both classic and viral sepsis (METosis, monocyte extracellular traps; TLR, Toll-like receptor; NLRP, NOD-like receptor protein; LPS, lipopolysaccharide; SARS-CoV-2, severe acute respiratory syndrome coronavirus-2; MyD88, myeloid differentiation primary response gene 88; NFkB, nuclear factor kappa-light-chain-enhancer of activated B cells)

We performed 93 MDW measurements on healthy blood samples before and after in vitro histone treatments. Firstly, based on the laboratory dataset on COVID-19 patients at hospital admission, we observed a mean MDW value of 25.58 ± 0.68, significantly higher compared to healthy subjects (*p* < 0.0001) (Fig. 1A).

These finding are in agreement with literature observations (reviewed in Ligi et al.) [17], and are mainly linked to monocyte hyperinflammatory activation characterizing COVID-19 illness [16, 18].

In our series of classical Sepsis patients MDW levels were found significantly higher compared to the values observed in both healthy subjects and COVID-19 patients (*p* < 0.0001) (Fig. 1A). Our results are in agreement with literature supporting the monocyte inflammatory processes in Sepsis patients caused by multiple bacteremia and associated with multiorgan failure and disease severity [6].

In our study, we treated in vitro whole blood samples with 100 µg/mL of a mixture of human histones to test the impact of histone levels found in critical COVID-19; similarly, we tested 100 µg/mL of histone mixtures + 1 µg/mL of LPS for studying Sepsis condition.

We demonstrated that healthy whole blood treated for 3 h with histone and histone + LPS showed MDW levels significantly higher compared to controls (*p* < 0.0001) (Fig. 1A). In particular, we found that histone-induced MDW values overlapped those found in COVID-19 patients with moderate/critical infection. Likewise, histone + LPS treatment results in a MDW increase similar to that found in Sepsis-affected patients (Fig. 1C).

A time-dependent increase of MDW induced by histone treatments was revealed (Fig. 1B). Furthermore, significant linear regressions sustained the time-dependency of MDW changes induced by histone (*Y* = 0.03751*x* + 18.06, *r*^2^ = 0.6995) and by histone + LPS (*Y* = 0.06951*x* + 17.85,* r*^2^ = 0.9317).

In our time-course studies, MDW value of controls did not significantly change at RT within 3 h (Fig. 1B). Furthermore, after 30 min of histone treatment, we revealed a significantly different MDW compared to respective controls (*p* = 0.0012), whereas histone + LPS showed an extremely significant difference vs control (*p* < 0.0001) (Fig. 1B). At this short time of treatment, no difference was found between histone + LPS and histone alone (Fig. 1B). After 60 min of incubation, a significant difference between histone + LPS vs histone alone (*p* = 0.0019) and between histone versus controls (*p* = 0.0065) were observed (Fig. 1B). After 3 h of incubation, extremely significant differences (*p* < 0.0001) were found among all treatments and vs controls (Fig. 1B).

No statistical difference was found in MDW values obtained between in vitro histone treatment and in vivo COVID-19 “*viral Sepsis”* infection; as well, no statistical difference between in vitro histone + LPS and in vivo bacterial/fungal “*classical Sepsis*” was observed (Fig. 1A, C).

## Discussion

For the first time, we demonstrated that histone concentrations similar to those found in critical COVID-19 condition [15], as well as in classical Sepsis [5, 6], are able to induce significant morphological modifications and a time-dependent increase of MDW value, related to monocyte heterogeneity and inflammatory activation [17], characteristics of both SARS-CoV-2 infection [18] and critical Sepsis condition [2, 11] (Fig. 1D).

Noteworthy, our in vitro whole blood experimental model demonstrated significant alterations of MDW values among treatments, but without significant modifications of both number and percentage of monocyte population up-to 3 h (data not shown). In full agreement with literature data, our results on histone-induced MDW modifications sustain the deleterious role of extracellular histones, which promote the monocyte-linked inflammatory processes, worsening the disease severity of both Sepsis and COVID-19 [2, 16].

The MDW index is based on specific positional parameters using simultaneously three independent energy sources: *direct current impedance,* to measure cell volume of cell types; *radio frequency opacity*, to characterize conductivity for internal composition of each cell; a *laser beam,* to measure light scatter for cytoplasmic granularity and nuclear structure [13]. The resulting MDW value quantitatively detects morphologic changes in reactive/activated monocyte cells, similarly to qualitative microscopic evaluation of a peripheral blood smear. In agreement with literature data, we found in healthy untreated controls a MDW index < 20.7 associated with normal morphological features of monocyte in blood smears; moreover, the homogeneity of monocyte populations in controls is highlighted through the innovative scatter plot (Fig. 1C, inset A). Comparing COVID-19 data with in vitro results of histone, we found similar scatter plots and overlapping MDW values, suggesting a closely associated monocyte heterogeneity (Fig. 1C, inset B vs. D), significantly different from controls. Likewise, in vitro histone + LPS treatments revealed scatter plots and MDW values overlapping to the features of in vivo Sepsis (Fig. 1C, inset C vs. E), extremely different from control values.

Interestingly, the comparison among controls vs histones vs histone + LPS revealed a significantly different profiles of monocyte heterogeneity, scatter plots and MDW values, sustained also by enhanced volume, intracellular vacuolization and granularity, membrane alterations and nuclear structure changes, as observed through blood smears (Fig. 1C, inset A vs. B vs. C).

Being up to 30–50% of Sepsis as culture negative, MDW and histone assay may provide additional clinical laboratory tools defining the classical and viral Sepsis conditions. Since these assays could not be hindered by possible limitations/bias (e.g., hemodilution; monocytopenic conditions; neither classic nor viral Sepsis showed low monocyte counts), both parameters may be routinely determined [7, 11].

Moreover, recent observations suggest possible therapeutic approaches with polyanions (e.g., heparins, heparinoids) [10] as potential strategies for protecting tissues from histone-induced inflammation/thrombosis [5, 19].

A possible limitation of our study may be linked to the lack of our analyses of histones in COVID-19 and Sepsis patients, due to the retrospective nature of this study.

Finally, although our findings were obtained in vitro in healthy subjects, we demonstrated that histones significantly affect monocytes, mechanistically acting as endogenous MDW modifiers and mirroring MDW features clinically observed during Sepsis (Fig. 1D).

Evaluations of further cellular/biochemical targets of histones (e.g., inflammatory and proteolytic pathways, and circulating blood proteins) in whole blood model is currently ongoing.

## Conclusions

We demonstrated that circulating histones represent one common mediator of monocyte alterations in both classic and viral Sepsis. We suggest MDW values and scatter plots as additional laboratory tools to simultaneously detect the monocyte volume, cytoplasmic granularity, and nuclear structure changes, paving the way for an early identification of enhanced monocyte heterogeneity in patients at higher risk of severe classical and viral Sepsis.

## Supplementary Information

**Additional file 1**: **Table S1** Comparison of MDW characteristics between healthy subjects after 3 h of in vitro treatment and in vivo patients affected by classic and viral Sepsis. **Table S2** Time-dependent MDW modifications obtained in healthy whole blood samples after in vitro treatments.

## Data Availability

The dataset used in this paper are not publicly available since they are still under elaboration for publication by the Authors but are available from the corresponding author upon request.

## Abbreviations

COVID-19: Coronavirus disease 2019
ET: Extracellular trap
LPS: Lipopolysaccharide
MDW: Monocyte distribution width
MOF: Multiorgan failure
SARS-CoV-2: Severe acute respiratory syndrome-CoronaVirus-2
