# Supplementary material for: Circulating histones contribute to monocyte and MDW alterations as common mediators in classical and COVID-19 sepsis
Source: Crit Care. 2022 Aug 30;26:260. doi: 10.1186/s13054-022-04138-2 (PMC9424804; doi:10.1186/s13054-022-04138-2)
Supplement: Supplementary file 1 — Additional file 1: Table S1 Comparison of MDW characteristics between healthy subjects after 3 h of in vitro treatment and in vivo patients affected by classic and viral Sepsis. Table S2 Time-dependent MDW modifications obtained in healthy whole blood samples after in vitro treatments. [file 13054_2022_4138_MOESM1_ESM.docx]

**ADDITIONAL FILE**

**Table S1: Comparison of MDW characteristics between healthy subjects after 3 h of *in vitro* treatment and *in vivo* patients affected by classic and viral Sepsis.**

|  | Mean±SD | Median | Min-Max | 25°-75° percentile | CV% | p |
| --- | --- | --- | --- | --- | --- | --- |
| CTRL 3h | 19.13±0.77 | 18.99 | 18.42 – 20.45 | 18.50 - 19.68 | 4.0 | --- |
| HISTONE | 24.37±1.97 | 23.52 | 22.43 – 27.31 | 22.82 - 26.36 | 8.1 | p < 0.0001 (vs. CTRL) |
| COVID-19 | 25.58±0.68 | 25.43 | 24.60 – 26.56 | 25.16 – 26.19 | 2.6 | p < 0.0001 (vs. CTRL)  n.s. (vs. HIS) |
| LPS+HISTONE | 29.68±1.51 | 29.27 | 28.44 – 31.74 | 28.50 - 31.27 | 5.1 | p < 0.0001 (vs. CTRL)  p < 0.0001 (vs. HIS) |
| SEPSIS | 30.40±0.80 | 30.39 | 29.29 - 31.41 | 29.63 – 31.17 | 2.6 | p < 0.0001 (vs. CTRL)  n.s. (vs. LPS+HIS)  p < 0.0001 (vs. COVID-19) |

*n.s. = Not significant*

**Table S2: Time-dependent MDW modifications obtained in healthy whole blood samples after *in vitro* treatments**

|  | Mean±SD | Median | Min-Max | 25°-75° percentile | CV% | p |
| --- | --- | --- | --- | --- | --- | --- |
| CTRL 0 min | 17.27±1.19 | 17.40 | 15.4 – 18.95 | 16.38 - 18.05 | 6.9 | --- |
| CTRL 30 min | 18.28±0.97 | 18.23 | 16.64 – 19.38 | 17.71 - 19.19 | 5.3 | n.s. (vs CTRL) |
| CTRL 60 min | 18.93±0.52 | 18.93 | 18.42 – 19.42 | 18.45 - 19.40 | 2.8 | n.s. (vs CTRL) |
| CTRL 180 min | 19.13±0.77 | 18.99 | 18.42 – 20.45 | 18.50 - 19.68 | 4.0 | n.s. (vs CTRL) |
| HISTONE 30 min | 21.53±1.69 | 21.80 | 19.07 – 23.12 | 20.08 - 23.01 | 7.8 | p < 0.0001 (vs CTRL) |
| HISTONE 60 min | 22.46±1.65 | 22.95 | 20.09 – 23.84 | 20.74 - 23.69 | 7.3 | p < 0.0001 (vs. CTRL)  n.s. (vs. HIS 30 min) |
| HISTONE 180 min | 24.37±1.97 | 23.52 | 22.43 – 27.31 | 22.82 - 26.36 | 8.1 | p < 0.0001 (vs. CTRL)  p = 0.0133 (vs. HIS 30 min)  n.s. (vs. HIS 60 min) |
| LPS+HISTONE 30 min | 23.11±1.27 | 23.33 | 21.49 – 24.27 | 21.80 - 24.19 | 5.5 | p < 0.0001 (vs CTRL) |
| LPS+HISTONE 60 min | 27.19±0.08 | 27.19 | 27.13 – 27.24 | 27.13 - 27.24 | 0.3 | p < 0.0001 (vs. CTRL)  p = 0.0129 (vs. LPS+HIS 30 min) |
| LPS+HISTONE 180 min | 29.68±1.51 | 29.27 | 28.44 – 31.74 | 28.50 - 31.27 | 5.1 | p < 0.0001 (vs. CTRL)  p < 0.0001 (vs. LPS+HIS 30 min)  n.s. (vs. LPS+HIS 60 min) |

*n.s. = Not significant*
